# New Concepts in Cancer Biomarkers: Circulating miRNAs in Liquid Biopsies

**DOI:** 10.3390/ijms17050627

**Published:** 2016-04-27

**Authors:** Erika Larrea, Carla Sole, Lorea Manterola, Ibai Goicoechea, María Armesto, María Arestin, María M. Caffarel, Angela M. Araujo, María Araiz, Marta Fernandez-Mercado, Charles H. Lawrie

**Affiliations:** 1 Molecular Oncology, Biodonostia Research Institute, 20014 San Sebastián, Spain; erika.larrea@biodonostia.org (E.L.); carla.sole@biodonostia.org (C.S.); lorea.manterola@biodonostia.org (L.M.); ibai.goicoechea@biodonostia.org (I.G.); maria.armesto@biodonostia.org (M.Arm.); maria.arestin@biodonostia.org (M.Are.); maria.caffarel@biodonostia.org (M.M.C.); angela.araujo@biodonostia.org (A.M.A.); marta.fernandez@biodonostia.org (M.F.-M.); 2 IKERBASQUE, Basque Foundation for Science, 48013 Bilbao, Spain; 3 Hematology Department, Donostia Hospital, 20014 San Sebastián, Spain; maria.araizramirez@osakidetza.eus; 4 Nuffield Department of Clinical Laboratory Sciences, University of Oxford, Oxford OX3 9DU, UK

**Keywords:** miRNAs, cfmiRNAs, liquid biopsies

## Abstract

The effective and efficient management of cancer patients relies upon early diagnosis and/or the monitoring of treatment, something that is often difficult to achieve using standard tissue biopsy techniques. Biological fluids such as blood hold great possibilities as a source of non-invasive cancer biomarkers that can act as surrogate markers to biopsy-based sampling. The non-invasive nature of these “liquid biopsies” ultimately means that cancer detection may be earlier and that the ability to monitor disease progression and/or treatment response represents a paradigm shift in the treatment of cancer patients. Below, we review one of the most promising classes of circulating cancer biomarkers: microRNAs (miRNAs). In particular, we will consider their history, the controversy surrounding their origin and biology, and, most importantly, the hurdles that remain to be overcome if they are really to become part of future clinical practice.

## 1. Introduction

Cancer represents the leading cause of morbidity and mortality worldwide, with approximately 14 million new cases and 8.2 million cancer related deaths in 2012, and this number is predicted to rise by approximately 70% over the next two decades according to the World Health Organization [1]. The effective and efficient management of cancer patients relies upon both early diagnosis and the frequent monitoring of patient response to treatment.

The current gold standard of cancer diagnosis is the histological examination of tissue, obtained either by radiologically guided biopsy or surgical excision. However, these procedures are invasive, expensive, and not without risk to the patient. They also take time and need to be consistently evaluated by expert pathologists. Therefore, there is a clear clinical need for alternative diagnostic techniques. In particular, the use of biological fluids such as blood as a source of non-invasive biomarkers of cancer has raised a great deal of interest [2]. So-called “liquidbiopsies” hold great clinical promise, as their non-invasive nature allows for rapid, economical, and repeat sampling—features that permit their use in screening programs and for the close monitoring of treatment response and disease progression, allowing for earlier intervention and dynamic treatment management. Furthermore, there is an increasing awareness of the genetic heterogeneity of tumors and a realization that tissue biopsies may miss this diversity. Liquid biopsies in contrast can capture the entire genetic panorama of the tumoral landscape. Consequently, this technology has the potential to radically improve current treatment regimens and therefore the outcome of cancer patients, allowing for a personalized approach to be taken for each patient. Although the majority of liquid biopsy research to date has focused upon the isolation of circulating tumor cells (CTCs), these cells are relatively rare and require sensitive collection and enrichment technology. Increasingly, the focus of liquid biopsy studies is shifting towards circulating (or cell-free) nucleic acids (cfDNA/RNA) as being easier to collect and analyze. There has been a particular interest in circulating cell-free microRNAs (cfmiRNAs), the subject of this review. For a wider overview of circulating nucleic acids as cancer biomarkers—in particular, mRNA and non-coding RNAs other than microRNAs (miRNAs)—we refer the reader to our previous article [2].

The history of circulating (blood) nucleic acids goes back to a finding in 1947 by Mandel and Metais of RNA and DNA in the plasma of healthy and sick individuals [3]. Remarkably, this report predates the realization that DNA was the molecule responsible for inheritance and the discovery of the double helix structure by Watson and Crick. It was not until the 1960s when scientific interest was aroused by the finding of cfDNA in patients with the autoimmune diseases, systemic lupus erythematosus (SLE) [4] and rheumatoid arthritis [5]. However, it was not until 1977 when the potential of cfDNA as cancer biomarkers was postulated—when Leon *et al.* reported elevated levels of circulating cfDNA in pancreatic cancer patients [6]. After that, in 1994, cancer-specific DNA mutations in NRAS (myelodysplastic syndrome (MDS)) [7] and KRAS (pancreatic cancer) [8] were found in the blood of cancer patients. cfRNA, in contrast to cfDNA, was not identified until 1999, when Lo *et al.* first identified (viral) cfRNA in the blood of nasopharyngeal carcinoma patients [9]. Several years later, in 2007, our group reported the presence of miRNAs in the blood of lymphoma patients [10]; the following year, it was demonstrated that miRNAs could be useful as non-invasive biomarkers of cancer [11,12].

**Figure 1:**
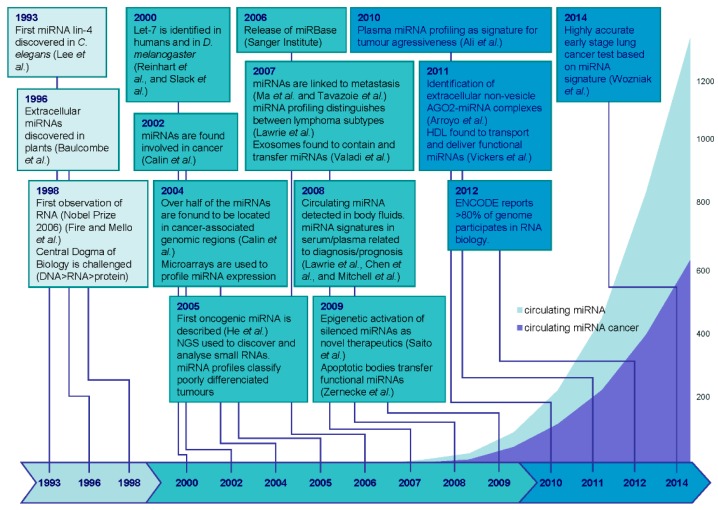
Chronological timeline of key discoveries in the microRNA (miRNA) field and their relevance to cancer. The overlapping plot depicts the number of PubMed-indexed publications for miRNA (dark blue) or miRNA related to cancer (light blue).

**Figure 2:**
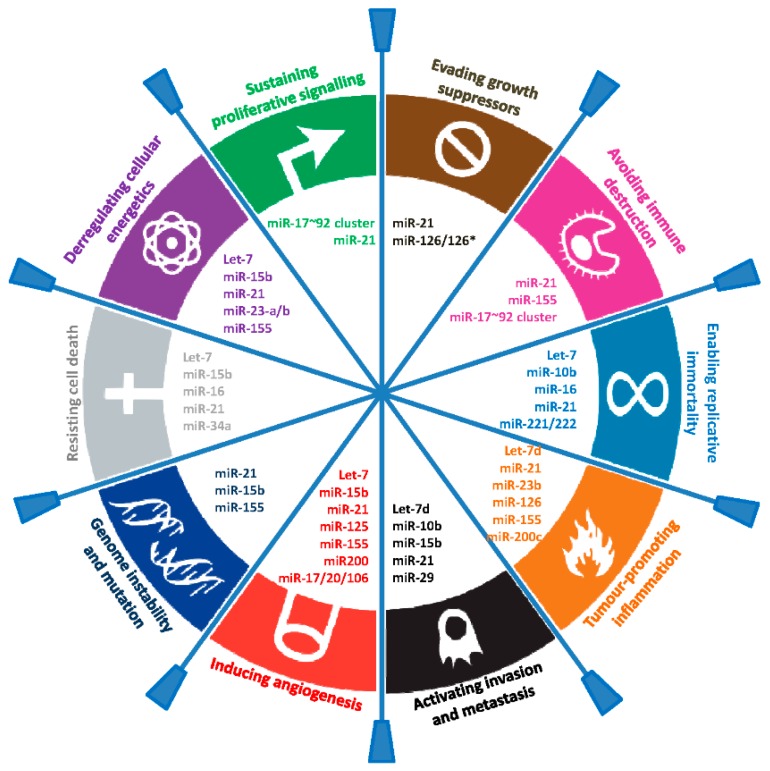
Selected circulating cell-free microRNAs (cfmiRNAs) and their functional role in the hallmarks of cancer.The figure lists some examples of biomarker cfmiRNAs (with a focus on the ones described in this review) that regulate genes involved in the different hallmarks of cancer as defined by Hanahan and Weinberg [18].

miRNAs are endogenous, small (18–24 nt), non-coding (nc) RNA molecules that regulate eukaryotic gene expression post-transcriptionally. miRNAs were unknown in science until just over 20 years ago, and, even then, were not formally recognized until 2001 [13]. There are now over 2500 human miRNAs that have been identified [14], and it is believed that nearly two thirds of all human genes are directly targeted by miRNAs [15]. miRNAs have been shown to play key regulatory roles in virtually every aspect of biology [16], including in the pathogenesis of cancer, and are aberrantly expressed in many diseases (Figure 1). Indeed, there is now compelling evidence that miRNAs regulate all aspects of the so-called “hallmarks of cancer” that enable tumor growth and metastatic dissemination [17,18] (Figure 2).

The field of circulating miRNAs has generated a great deal of interest and has been growing at an exponential rate with more than 2000 publications now published on the subject (source: PubMed; Figure 1), and many conferences and commercial entities are involved in this area. Below, we discuss some of the controversies behind the origin of these molecules and their possible functions. We also review some of the major evidence to suggest their potential as cancer biomarkers, but, most importantly, we discuss some the barriers that are still to be overcome if these molecules are to become a part of routine clinical practice.

### 1.1. Origin of Extracellular miRNAs

There are several different hypotheses that have been proposed to explain the presence of circulating miRNAs in biological fluids [19,20,21]. These include the passive release of miRNAs from broken cells after tissue injury, cell apoptosis or necrosis, chronic inflammation, and from cells with a short half-life such as platelets [22,23,24]. For example, specific miRNAs are elevated in blood after myocardial infarction [24,25,26] or hepatobiliar injury [27]. An alternative hypothesis, though not mutually exclusive, is that miRNAs are actively secreted from cells either shuttled via microvesicles such as exosomes or shedding vesicles [12,28,29,30], or directly in complex with RNA-binding proteins or lipoproteins such as nucleophosmin (NPM1) [31], high-density lipoprotein (HDL) [32], or Argonaute proteins [22,33].

There is some controversy as to which of these represent the true origin of cfmiRNA, or at least the relative proportion of the different routes; until fairly recently, it was believed that most circulating miRNAs were derived from cell-derived vesicles [34].This has been contested by at least two independent reports that suggest that more than 90% of the miRNAs in blood are membrane-free and associated with Ago proteins [22,33]. Irrespective of their origin, the composition of cfmiRNAs appears to differ greatly from their respective donor cells [35]. In fact, some secreted miRNAs are notpresent at all in the parental cells [30].

### 1.2. Cell–Cell Communication (Hormone-Like Molecules)

Aside from their (passive) role as biomarkers, there has been a great deal of interest in the function of cfmiRNAs and in particular their ability to act as signaling molecules that potentially allow tumor cells to modify the bodies response to its own advantage. The first evidence that extracellular miRNAs could act as signaling molecules was discovered in plants in 1996 [36]. There is now emerging evidence of human miRNAs acting in a similar fashion either as paracrine signalers or even as systemic communicators between cells in an endocrine manner (in a hormone-like way) [20,37]. A number of facts support this possibility: miRNAs appear to be selectively packaged and secreted [31,38]; extracellular miRNAs are protected from RNases either by lipoprotein or protein carriers or by microvesicle membranes [33]; and circulating miRNAs are able to alter gene expression in recipient cells and mediate functional changes in them [30,35,39,40]. The first indication that miRNAs could shuttle between cells via exosomes was demonstrated in mast cells [29]. Later, the transfer of miRNAS between different cell types (embryonic stem cells and fibroblasts) was demonstrated [41]. More recently, exosomal miRNA has been shown to be able to modulate inmunological response through modification of the gene expression of antigen presenting cells (APC) by T-cells, B-cells, and dendritic cell-derived miRNAs [35].

Multiple studies suggest that cfmiRNAs could play a role in cancer biology through tumor-derived exosomal miRNA modulating non-tumor cells to the ultimate benefit of the tumor. For example, exosomal-cfmiRNAs have been demonstrated to modulate chemosensitivity [42], angiogenesis, and cell invasiveness [43,44,45,46]. While this is potentially a fascinating phenomena, this is still a contentious issue, and it is worth remembering that the few studies carried out to date have been almost exclusively *in vitro*. Finally, although Ago2-boundmiRNAs appear to form the majority of cfmiRNA, there is no evidence (or known mechanism) for the active release of vesicle-free AGO2-miRNA complexes in mammals, nor any indication of Ago-2 surface receptors for the uptake by recipient cells. Therefore, the physiological relevance of cfmiRNA as an intercellular signaling mechanism remains to be determined.

## 2. miRNAs as Cancer Biomarkers

According to the National Cancer Institute, a biomarker is defined as “a biological molecule found in blood, other body fluids, or tissues that are a sign of a normal or abnormal process, or of a condition or disease.” In cancer, they can be divided into three general categories: (1) diagnostic biomarkers, which are used for a differential diagnosis; (2) prognostic biomarkers, which can distinguish tumors with a good outcome from those with a bad outcome; and (3) predictive biomarkers, which are for assessing whether a treatment is likely to be effective for a particular patient or not. An ideal biomarker should have a high specificity, sensitivity, and predictive power. miRNAs have a number of intrinsic characteristics that make them attractive as biomarkers. Firstly, they are highly specific, and it has been shown that miRNA expression profiles differ between cancer types according to diagnosis and the developmental stage of the tumor, with a greater resolution than traditional gene expression analysis [47]. Secondly, unlike other RNA classes, miRNAs are remarkably stable and therefore can be robustly measured not only in biological fluids but also from routinely prepared formalin-fixed paraffin-embedded (FFPE) material [48]. Indeed, unlike other RNA species, miRNAs appear resistant to boiling, pH changes, repeated freeze-thawing cycles, and fragmentation by chemical or enzymes [12,20,49]. It should be noted, however, that cfmiRNAs are not themselves intrinsically resilient to RNase or any other treatment; rather, they are protected by their lipidic or protein-based carrier [12,50,51]. As a result of these characteristics, the use of cfmiRNAs as biomarkers—and in particular as cancer biomarkers—has generated a plethora of publications over the last few years. Due to the limitations of space, we will not attempt to review all of these but instead discuss the more robust studies that identify common cfmiRNA biomarkers in multiple studies. More often than not, these biomarker miRNAs are themselves intimately involved in cancer pathology, as shown in Table 1, which includes their respective experimentally validated targets. While it may be tempting to speculate that these miRNAs may have the same effect while in circulation as intracellularly, there is no evidence that this is indeed the case.

### 2.1. let-7 Family (let-7a, -7b, -7c, -7e, -7f, -7i)

There are 13 different *let-7* family members in humans: *let-7a-1*, *7a-2*, *7a-3*, *7b*, *7c*, *7d*, *7e*, *7f-1*, *7f-2*, *7g*, *7i*, *miR-98*, and *miR-202* [244]. Differential expression of *let-7* family members has been described to be downregulated in a wide variety of cancers such as melanoma, pancreatic cancer, prostate cancer, and sarcoma, although some, including lymphoma, mesothelioma, and breast cancer, have been shown to be upregulated; thus, the *let-7* family is generally regarded as a tumor suppressor [245]. *let-7* has been shown to be a direct regulator of some important oncogenes, such as the three *RAS* genes [246,247], *HMGA2* [248,249], *STAT3* [250], *UHRF2* [251], and *MYC* [252,253,254]; additionally, *let-7* family targets cell cycle and cell proliferation genes [255,256,257]; finally, apoptosis is also shown to be regulated by *let-7* family, through *CASP3* targeting [258]. RNase III nuclease, known to process pre-miRs, was also confirmed as a direct target of the *let-7* family, so they might regulate their own processing [259,260].

*let-7* family members have been identified differentially expressed and therefore have been proposed as diagnostic tools in serum/plasma of many cancer types including lung cancer (*let-7a*, *let-7c*, *let-7f*) [57,60,67], prostate cancer (*let-7a*) [52], gastric cancer (*let-7a*, *let-7c*, *let-7i*, *let-7f*) [56,62], ovarian cancer (*let-7b*, *let-7f*, *let-7i*) [59,66,70], hepatocellular carcinoma (HCC) (*let-7b*, *let-7f*) [58,68], breast cancer (*let-7c*, *let-7b*, *let-7g*) [61,261], acute myeloid leukemia (AML) (*let-7b*, *let-7d*) [262], thyroid carcinoma (*let-7e*) [65], and colorectal cancer (CRC) (*let-7a*) [53,54]. Recently, *let-7f* has also been detected as deregulated in the feces of CRC patients [54]. However, their potential as prognostic biomarkers has also been highlighted in several cancer types such as myelodisplasia (*let-7a*) [55], lung cancer (*let-7b*, *let-7f*, *let-7i*) [57,67,69], hepatocellular carcinoma (*let-7b*, *let-7f*) [58,68], multiple myeloma (*let-7e*) [64], prostate cancer (*let-7d*) [63], ovarian cancer (*let-7f*) [66], and breast cancer (*miR-202*) [263]. In general, low *let-7* levels are associated with poor prognosis including overall survival, early recurrence, and tumor size.

### 2.2. miR-10b

*miR-10b* acts as a metastasis driver in many different types of cancers such as breast cancer [264], glioma [265], and oesophageal cancer [266], among others, specifically promoting cell mobility and invasiveness. Validated targets of this miRNA include *SDC1* [267], *HOXD10* [264,265,268,269], *KLF4* [266,270], *MICB* [271], and *CDH1* [272]. It also regulates *E2F1*-mediated transcription through *p21/CDKN1A* regulation [273] and important cell cycle regulators such as *BUB1*, *PLK1*, and *CCNA2* [274].

Circulating *miR-10b* levels have been described as being upregulated in patients with ovarian cancer [30], lung cancer [29], oesophageal [75], and glioblastoma [73] compared to healthy controls. Consistent with this, *miR-10b* is increased in plasma from metastatic breast cancer patients [27,28] and in the cerebrospinal fluid of patients with brain metastasis of both breast and lung cancer [73].

### 2.3. miR-16

*miR-16* has an important role in regulating apoptosis in different cancer types including lung, breast, liver, glioblastoma, and squamous cell carcinoma through targeting *FEAT/METTL13* [275], *RPS6KB1*, *IGF1R* [276], *CCND1* [277], *BCL2* [278], *RECK*, and/or *SOX6* [279]. This miRNA is also an important regulator of cell cycle molecules including *FG2F*, *CCNE1*, and *E2F1* [280,281,282], as well as cell autophagy (*mTORC2*) and metastasis (*SOX5*) [283,284].

Circulating *miR-16* has been described significantly differentially expressed in patients compared with healthy controls in several cancers: oral cancer [76], breast cancer [79], prostate cancer [36], osteosarcoma [80], gastric cancer [81,82], liver carcinoma [68], and oesophageal carcinoma [83]. Furthermore, *miR-16* is also associated with prognosis and tumor size in gastric cancer [81,82], hepatocellular carcinoma [68], and esophageal squamous cell carcinoma (ESCC) [83].

### 2.4. The miR-17~92 Cluster

Over-expression of the *miR-17~92* cluster is a key oncogenic event in many cancer types, and overexpression in murine models result in tumor formation. This cluster is composed of different miRNAs: *miR-17*, *miR-18a*, *miR-19a*, *miR-19b*, *miR-20a*, *miR-92*, and *miR-106a/b* with a variety of related functions, primarily targeting tumor suppressor molecules and pathways such as *PTEN* and *RB1* [285,286], and molecules in the TGFβ signaling pathway such as *TGFBR2*, *SMAD2*, and *SMAD4* [287,288,289]. These miRNAs also target senescence (*p21/CDKN1A*) [290,291], metastasis (*DLC1* and *TIMP2*) [292,293,294], cell cycle regulation (*E2F* family members, *RB1* and *p21*/*CDKN1A*), and angiogenesis (*THBS1* and *CTGF*) [295,296,297].

Circulating members of the *miR-17~92* cluster have been widely described as being deregulated in many cancer types including colorectal cancer [56,87,92,98,101,103,105,106,107,231,298], gastric cancer [56,81,85,109,113], squamous cell carcinoma [84], breast cancer [86,90,114], bladder cancer [91], hematological malignancies [93,104,117,202,299,300], lung cancer [94,95,97], prostate cancer [77], osteosarcoma [80], oesophageal carcinoma [99], ovarian cancer [81,115], and hepatocellular carcinoma [110,111,301]. They have also been shown to have prognostic value in colorectal cancer [92,100,108], sporadic melanoma [88], breast cancer [71,112], bladder cancer [91], multiple mieloma [93,104,299,300], lung cancer [94], and prostate cancer [96]. Moreover, levels of circulating *miR-17~92* miRNAs have been associated with the response to chemotherapy (*i.e.*, predictive biomarkers) in both breast cancer [89] and multiple myeloma [93].

### 2.5. miR-21

*miR-21* acts mainly as an oncogene (“onco-miR”) because most of its target genes are tumor suppressors. The list of these target genes is extensive, and they are related to all hallmarks of cancer [302]. One of the principal *miR-21* targets is *PDCD4*, which is a tumor suppressor gene that inhibits PMA-induced neoplastic transformation [303], tumor promotion and progression [304], and invasion and intravasation [305]. *miR-21* targets multiple components of *TP53*, *TGFB1*, and mitochondrial apoptosis tumor suppressive pathways (including *HNRPK* and *TP63*) [306]. Other targets of *miR-21* have been related mainly with apoptosis, cell growth, migration, and invasion, such as *BCL2* [307], *PTEN* [307,308,309], *RECK* [310], *RHOB* [311], and *TPM1* [312], among others.

Circulating *miR-21* has been described in a lot of different cancers as a diagnostic, predictive, and/or prognosis biomarker. Some of these are hematological cancers [116,117], breast cancer [124,125], gastric cancer [128,130], ovarian cancer [134,135], pancreatic cancer [136,141], colorectal cancer [100,145], lung cancer [97,149], and liver cancer [155,156], among others.

### 2.6. The miR-29 Family (miR-29a, -29b, and -29c)

The *miR-29* family members act as tumor suppressors, and their downregulation is associated with many cancer types including leukemia [313,314,315], melanoma [316], liver cancer [317,318], colon cancer [319], cervical cancer [320], lung cancer [321], and prostate cancer [322]. In many studies, downregulation of *miR-29* has correlated with more aggressive forms of cancer and shorter overall survival [316,321,323]. It has been demonstrated to directly target genes involved in the control of the cell cycle (*CDK6*) [319,320,323] and apoptosis (*MCL1*, *BCL2* and *FHIT*) [315,319,321], as well as genes that promote cell migration and invasion (*LAMC1*, *CDC42*) [322,324]. Furthermore, the *miR-29* family target genes such as *PIK3R1* and *CDC42* that normally suppress *TP53* [324].

Differential expression of *miR-29* family members in plasma/serum has been observed in several cancer types. The expression levels of all the *miR-29* family members were upregulated in sera of patients with osteosarcoma. In particular, *miR-29a* and *miR-29b* were associated with poor prognosis [162]. *miR-29a* has been shown to be upregulated in colorectal cancer and therefore has been proposed as a potential non-invasive biomarker for early detection of colorectal cancer [101,103,164,231], also involving liver metastasis [163]. It has also been found to be upregulated in breast cancer [165,166] and downregulated in oral and ovarian cancer, compared with healthy controls [76,102]. Similarly, serum levels of *miR-29b* have been proposed as potential biomarkers for diagnosis and prognosis of colorectal cancer [167], whereas *miR-29c* could be useful as a predictor of postoperative early relapse [168]. However, it was found to be downregulated in serum of nasopharyngeal carcinoma patients, compared with controls [170].

### 2.7. The miR-30 Family (miR-30a, -30b, -30c, -30d, and -30e)

Similar to the *miR-29* family, *miR-30* family members appear to act primarily as tumor suppressors in several cancer types such as ovarian cancer, breast cancer, non-small cell lung cancer (NSCLC), and colorectal carcinoma [325,326,327,328,329,330], although they have also been reported as oncogenes [331]. Several genes have been described to be regulated by the *miR-30* family, such as some epithelial-to-mesenchymal transition (*EMT*)-associated genes [332], anti-apoptotic protein *AVEN* [333], and *DLL4* which has a fundamental role in angiogenesis [334].

Members of the *miR-30* family have been identified differentially regulated in body fluids, but their potential as biomarkers has mostly been reported in combinations with other miRNAs. For example, a blood test based upon a combination of the levels of five miRNAs including *miR-30c* has been described to effectively differentiate prostate cancer patients from benign prostatic hyperplasia (BPH) patients and healthy controls [173]. In addition, the combination of four plasma circulating miRNAs, including *miR-30c* and serum *PSA*, has a greater potential to be used as a noninvasive diagnostic biomarker for prostate cancer screening than *PSA* testing alone [174]. Similar studies have been reported for *miR-30c* and *miR-30a-3p* in lung adenocarcinoma [171,175], *miR-30a* in esophageal adenocarcinoma [172], and *miR-30d* in lung cancer [176]. In hepatocellular carcinoma, *miR-30e* has been recently found to be downregulated in serum when compared with healthy controls [178], and, in lung cancer, high levels of *miR-30d* in serum have been associated with a shorter overall survival [176,177].

### 2.8. The miR-34 Family (miR-34a, -34b, and -34c)

Members of the *miR-34* family are well known to regulate cell cycle, senescence, apoptosis, and invasiveness in cancer, and deregulation of *miR-34a* has been reported in several types of cancers [335,336]. The *miR-34* family targets multiple TP53 inhibitor genes (*MDM4*, *SIRT1*, *MTA2*, *HDAC1*, and *YY1*) and promotes proliferation arrest and induction of apoptosis by targeting *MYC*, *CDK6*, and *MET*. These genes encode factors required for G1/S transition (*MYC*, *E2F*, *CDK4*, and *CDK6*), anti-apoptotic proteins (*BCL2*, *SIRT1*), and proteins involved in invasion (*MET*) [337]. It has also been reported to target pluripotency genes such as *NANOG*, *SOX2*, and *MYCN* [338,339] and components of Wnt signaling pathways [340,341] and notch signaling pathways [342,343], which regulate growth, epithelial–mesenchymal transition (EMT), and metastasis.

Elevated levels of *miR-34a* in serum can discriminate between breast cancer patients and healthy controls, and are also associated with the presence of overt metastasis [72,237]. High levels of circulating *miR-34a* have also been observed in ovarian and lung cancer [72,74,179], and *miR-34b* has been found to be upregulated in serum from prostate cancer patients [77]. In osteosarcoma patients, *miR-34b* levels were found to be downregulated when compared with controls, and these expression levels were significantly decreased in the metastatic patients [180]. Similarly, downregulation of circulating *miR-34c* in serum of NSCLC patients and *miR-34b/c* in serum of breast cancer patients has been reported and might have potential as biomarkers for the diagnosis of these pathologies [181,182].

### 2.9. The miR-125 Family (miR-125a and -125b)

*miR-125* has been shown to act as a tumor-suppressor in several cancers including ovarian cancer [313,344], bladder cancer [345], breast cancer [346,347], hepatocellular carcinoma [348,349,350], melanoma [351], cutaneous squamous cell carcinoma [352], and osteosarcoma [353]. *miR-125* targets several genes associated with carcinogenesis such as transcription factors (*STAT3* and *E2F3*) [345,353], matrix-metalloprotease (*MMP11* and *MMP13*) [348,352], members of the *BCL2* family [354,355], and growth factors (*VEGFA*) [348].

Deregulated levels of *miR-125a* were present in the saliva of oral squamous cell carcinoma (OSCC) patients and in serum of NSCLC patients compared with healthy controls [183,184]. In a similar way, *miR-125b* levels were significantly lower in glioma patients and in serum-derived exosomes of melanoma patients [192,193]. In addition, low circulating levels of *miR-125a* have been associated with poor prognosis in both breast cancer and hepatocellular carcinoma [185,186]. In contrast, *miR-125b* was found to be upregulated in the plasma and serum of metastatic prostate cancer patients [356], breast cancer [125], OSCC [190], colorectal cancer [191], and NSCLC [188], in comparison with healthy controls, and to be associated with poor prognostic outcome and chemotherapeutic resistance in this cancer [187,188,189].

### 2.10. miR-155

*miR-155* is involved in both physiological (hematopoiesis andimmune response) and pathological processes. The oncogenic role of *miR-155* is well established in both hematological malignances as well as solid cancers such as breast cancer, where its overexpression is generally correlated with poor prognosis [357,358]. Validated *miR-155* target genes are present in multiple pathways associated with cancer and cancer progression, including *EMT* (*SMAD5*), proliferation (*SOCS1*, *INPP5D*, and *CSF1R*), block of differentiation (*SPI1*, *CEBPB*), and apoptosis (*CASP3*, *FADD*, *APAF1*, and *FOXO3A*) [359,360,361,362,363,364,365,366,367].

In many studies, differentially expressed levels of circulating *miR-155* have been identified, including breast cancer [90,194,195,196], colorectal cancer [198], lung cancer [57,199], AML [201], diffuse large B-cell lymphoma (DLBCL) [11,202], and esophageal cancer [200], making it a potential non-invasive diagnostic biomarker for early detections in these pathologies. In fact, a biosensor for *miR-155* detection in plasma has recently been developed for the diagnosis of breast cancer [368]. Elevated levels of *miR-155* are also related to overt metastasis in breast cancer [72,197], and these high levels have also been identified not only in blood but also in the urine of breast cancer patients [126]. In addition, *miR-155* has been also suggested as a prognostic biomarker in c*hronic lymphocytic* leukemia (CLL) and adult T-cell leukemia (ATL) [203,204], and as a predictive biomarker to response to therapy in CLL [203].

### 2.11. The miR-200 Family (miR-200a, -200b, -200c, -141, and -429)

The *miR-200* family is believed to play crucial roles in both cancer initiation and metastasis—in particular, in epithelial-mesenchymal transition (EMT)—primarily through the targeting of *ZEB1* and *ZEB2* transcription factors [369,370]. It has also been associated with angiogenesis by the targeting of *VEGFA* and *VEGF* receptors [371,372] and pro-angiogenic ligands such as *CXCL8* and *CXCL1* [373].

Elevated serum levels of *miR-200a*, *miR-200b*, *miR-200c*, and *miR-141* have been suggested as good biomarkers for diagnosis and prognosis in ovarian cancer [205,206,208], and serum levels of *miR-429* were associated with poor overall survival in NSCLC [169]. In addition, elevated levels of circulating *miR-141* have been identified to show diagnostic potential in patients with upper urinary tract urothelial cancer [216], lung cancer [74], prostate cancer [12,217,218,219,220], breast cancer [207], and bladder cancer [374]. In breast and bladder cancer, this upregulation is also associated with prognosis. Furthermore, *miR-200c* was found significantly elevated in the plasma of patients with colorectal cancer [210], gastric cancer [212,213], and breast cancer [207], and this upregulation was associated with poor prognosis. In colorectal cancer, it has also been identified as a metastasis predictive biomarker [211] as well as *miR-141* [221]. Similarly, *miR-200c* can be useful to predict prognosis in NSCLC [214] and in esophageal cancer. In the latest research, the serum level of *miR-200c*, as well as *miR-200b*, can be useful for predicting response to chemotherapy [215], and also has prognostic value in prostate cancer and predictive value in docetaxel chemotherapy outcomes [209].

### 2.12. miR-210

*miR-210* is strongly linked with the hypoxic pathway and angiogenesis through the targeting of *EFNA3* [375,376], *VEGF* [377], and *STAT3* [378]. *miR-210* also acts upon cell cycle and apoptotic pathways by targeting *E2F3*, *MNT* [379,380,381], *FGFRL1* [382], *BCL2* [383,384], and *STAT3* [378]. Furthermore, *miR-210* can inhibit DNA damage repair genes such as *RAD52* [385] and oncogenes such as *HOXA1* [386].

Circulating *miR-210* levels have been shown to have diagnostic value in DLBCL [11], pancreatic cancer [136,141,142,222,226], bladder cancer [227,228], glioma [229], liver carcinoma [230], and renal carcinoma [223,224] and with the presence of metastasis in patients with breast cancer [225] and pancreatic cancer [226]. *miR-210* has been also correlated with sensitivity to treatment in breast cancer and with prognosis in patients with breast cancer [225], pancreatic cancer [226], bladder cancer [227], and liver carcinoma [230].

### 2.13. miR-221/-222

*miR-221* and its paralogue *miR-222* are known to target angiogenesis by direct interaction with *KIT* [387,388], *PTEN* [389], *TIMP3*, *ADAM10*, and *ADAM17* [390] and by indirectly regulating endothelial nitric oxide synthase expression [387,391]. *miR-221/-222* have also been described as regulators of cell proliferation via the targeting of *SEMA3B* [392], *IRF2*, *SOCS3* [393], *p27*/*CDKN1B* [394,395], *HECTD2*, *RAB1A* [396], *β-catenin*/*CTNNB1*, *TGFB1* [397], *ADAM17*, *ITGB4*, and *STAT3* [398]. Other pathways regulated by *miR-221/-222* include apoptosis and metastasis via PTEN [389], IRF2, SOCS3 [393], BBC3 [399], SEMA3B [392], HECTD2, RAB1A [396], ADAM17, ITGB4, STAT5A [398], and Ecm29/KIAA0368 [400]; in resistance to chemotherapy in some type of cancers through PTEN [389] and β-catenin/CTNNB1 [397] regulation.

Circulating *miR-221/-222* levels have been identified as diagnostic markers in prostate cancer [96], colorectal carcinoma [87,231], NK/T-cell lymphoma [104], liver carcinoma [111], larynx cancer [233], glioma [234], and melanoma [235]. They also have prognostic value in glioma [234], melanoma [235], prostate cancer [96], and renal carcinoma [236].

### 2.14. miR-375

*miR-375* is a tumor suppressor miRNA that has been described in different kind of cancers, where it targets genes related to proliferation and apoptosis (*JAK2*, *PDK1*, *14-3-3ζ*, *IGF1R*, *KLF4*, *KLF5*, *survivin*, *ERBB2*, *PIK3CA*, *MTDH*, *YAP1*, *CIP2A*/*KIAA1524*, *MTDH*, and *BCL2*) [401,402,403,404,405,406,407,408,409,410,411,412,413,414] as well as metastasis (*IGF1R*, *CLDN1*, *CIP2A*/*KIAA1524*, and *BCL2*) [404,412,414,415] and mediates resistance to therapy (*IGF1R*, *TP53*, and *PHLPP1*) [416,417,418]. Furthermore, *miR-375* is involved in epithelial to mesenchymal transition in breast cancer [419] and targets *ATG7* inhibiting autophagy and impairing the viability of cells under hypoxic conditions in liver cancer [420].

Circulating *miR-375* has been identified as a diagnostic biomarker in oesophageal carcinoma [83,153], liver cancer [242], colorectal cancer [243], and lung cancer [421]. Additionally, in prostate cancer [218,239,240], lung cancer [238], and oesophageal carcinoma [241], *miR-375* has been shown to have prognostic value.

## 3. Extracellular miRNAs in Other Biological Fluids

**Figure 3:**
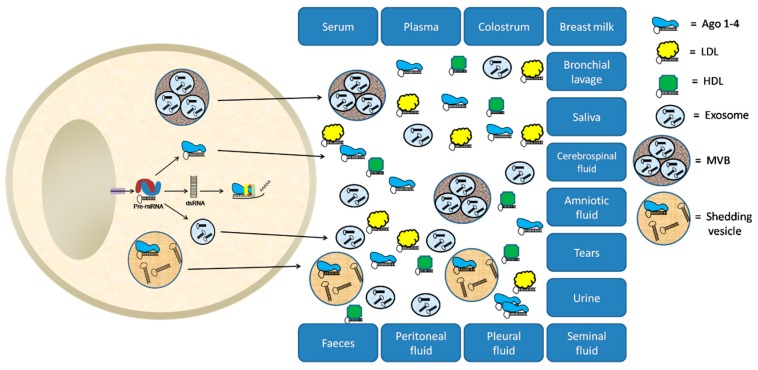
Origin of extracellular RNA. Several hypotheses have been proposed to explain the presence of miRNA in biological fluids, including the passive release of miRNA from broken cells and tissues and the active secretion from cells in microvesicles or conjugated to RNA-binding proteins. Cell-free miRNA can be detected in different body fluids including plasma, serum, saliva, tears, urine, amniotic fluid, colostrum, breast milk, bronchial lavage, cerebrospinal fluid, peritoneal fluid, pleural fluid, and seminal fluid and also in feces. Ago 1–4: Argonaute proteins 1–4; LDL: Low-density lipoprotein; HDL: High-density lipoprotein; MVB: Multivesicular body.

In addition to blood, other biological fluids such as urine, saliva, cerebrospinal fluid, vitreous humor of the eye, breast milk, seminal fluid, and tears have been studied as potential sources of miRNA biomarkers [422,423] (Figure 3). The majority of these studies concern tumor types associated with the source of the biological fluids. For example, saliva has been studied in head and neck squamous cell carcinoma [183,424,425,426,427], tumors of the parotid gland [183,424,428], esophageal cancer [154], and pancreatic cancer [142,429]. Urine is another well studied source of cfmiRNAs associated with cancer in particular urological cancers including prostate and bladder cancer (reviewed in [430]). In addition, several studies have looked at the potential of urine for miRNA biomarkers in ovarian, breast, and liver cancer [126,431,432]. miRNAs in cerebrospinal fluid have been described as potential biomarkers for the diagnosis and monitoring of disease in brain tumors such as glioblastoma but also in CNS lymphomas and in brain metastases of non-neuronal origin [73,433,434,435,436]. In a similar vein, miRNAs, in the vitreous humor of the eye, have been identified in ocular cancers including vitreoretinal lymphoma or uveal melanoma [437,438]. It also has been suggested that the miRNA profile of breast milk could be a more sensitive biomarker for breast cancer than blood-associated miRNAs [439] and that seminal fluid-associated miRNAs can serve as biomarkers of prostate cancer [440].

## 4. Discussion

### Challenges in Studying cfmiRNA

A major obstacle to the translation of cfmiRNAs from laboratory studies into the clinic is the lack of consistent and robust results with many apparently contradictory reports in the literature. A likely reason for this lack of reproducibility is that there are very few multi-center studies, and cohorts are often insufficiently powered. Another confounding factor is the fact that there is a high degree of inter-individual variability in the levels of cfmiRNAs, even when focusing only on healthy populations [441]. Moreover, there is a technical source of variation between studies, such as the starting material used for the experiments (e.g., the purification of cells, the cell types, the control populations used, the RNA extraction method, *etc.*), the technological platforms (e.g., microarray, qRT-PCR *vs.* next generation sequencing (NGS) *etc.*), and the differing statistical methodologies used.

The blood collection and processing represent critical points of variability in cfmiRNA studies. In the first instance, miRNA contamination can occur at the venopuncture site itself [442]. After extraction, the elapsed time between blood collection and processing should be minimized to prevent lysis and cellular contamination, which can be a major source of variability between samples [443,444,445,446]. In addition, the choice of anti-coagulant used in plasma collection can influence downstream detection technologies, such as qRT-PCR and heparin-coated tubes, should be avoided [447]. Another major source of difference in cfmiRNA profiles comes from the choice of whether to use serum or plasma, and whether to purify exosomes or use whole serum/plasma [448,449,450,451].

The choice of RNA purification procedure can also critically affect the results of cfmiRNA studies and should be considered carefully in terms of experimental design. For example, small RNA molecules with low GC content are known to be selectively lost during Trizol-based extraction (the most popular method) when present in low concentrations, such as in biological fluids, and thus should be avoided if possible; specific commercially available kits should be used instead [452]. Many researchers use non-human miRNAs (e.g., *C. elegans* sequences) as spike-in controls to control for variability between the miRNA extraction efficiency between samples [12]. Another important issue is that it is almost impossible to accurately quantify RNA in samples from biological fluids due to the low quantities of RNA present and the high levels of contaminating salts and protein that can interfere with spectrophotometric measurement. Therefore, studies often use fixed volumes of plasma to standardize, even if it is evident that they may contain different amounts of RNA [453].

**Figure 4:**
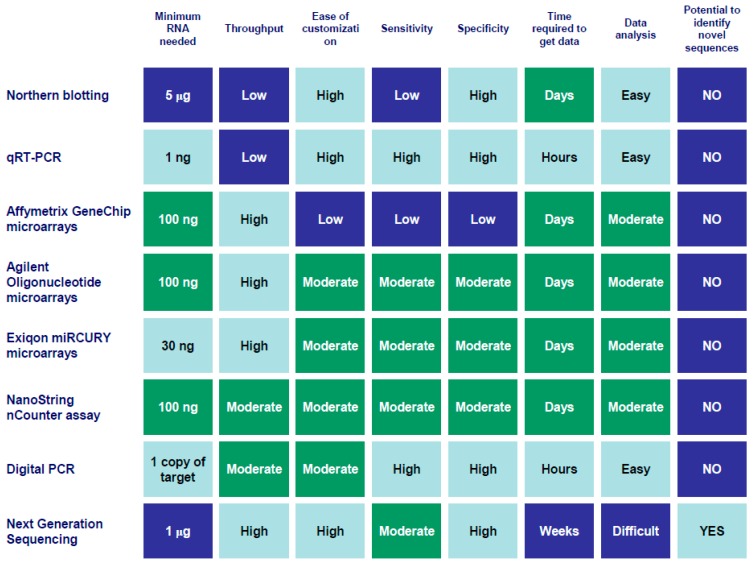
Comparison of methods commonly used to study extracellular RNA. Color code indicates the relative feasibility of that particular technique based on a given feature, from light blue (more feasible), through turquoise, to dark blue (less feasible). Data analysis: Easy (feasible in any molecular biology lab), Moderate (various software platforms available), Difficult (requires advanced computational infrastructure).

There are many different methods available to measure cfmiRNAs, including qRT-PCR (LNA-based, Taqman or other proprietary technologies), digital PCR (dPCR), microarrays, and next generation sequencing (NGS) techniques. The choice of platform depends largely on the experimental design required (Figure 4). Importantly, it should be borne in mind that the choice of technique can massively influence measurements; indeed, several studies show a lack of concordance between platforms when using the same sample source [454,455].

Another challenging issue in cfmiRNA studies is the lack of consensus about a suitable endogenous reference to use in biological fluids, as the small nucleolar RNAs (snoRNAs) generally used as reference genes in miRNA cell-based studies is not present in biological fluids due to degradation [456,457]. As an alternative, individual miRNAs themselves are frequently used [11,12,458]. However, it has been shown that the expression levels of the most commonly used housekeeping miRNAs in cfmiRNA studies vary significantly between samples depending upon the pathology that is being studied [77,459]. Therefore, miRNAs to be used as reference genes have to be chosen with care, determining empirically for each experiment which miRNAs are more stable (using geNorm and/or NormFinder algorithms), an approach taken by some studies [458,459] but not always possible when sample volumes are limited. Alternatively, a more economical option is to include at least two (preferably three) miRNAs as reference controls for cfmiRNAs studies.

## 5. Conclusions

It is clear that there is a great deal of interest in liquid biopsies, and in cfmiRNA in particular, as a viable alternative to tissue-based sampling in the clinic. Such an approach would bring a fundamental change to cancer patient management by allowing repeated sampling for treatment response monitoring, an assessment of tumor heterogeneity, and even cancer screening programs. cfmiRNAs are particularly attractive candidates for non-invasive cancer biomarkers due to their surprising degree of stability in biological fluids; as we have outlined above, there is now a wealth of literature to suggest that this class of molecules holds great clinical promise. The caveat is that, as a very recently discovered field, there appears to be little agreement between seemingly identical studies, presumably due to many different factors outlined above between studies. In others words, there is a clear need of setting standardized approaches to be put into practicein future cfmiRNA biomarker studies if these molecules are to ever make their way into routine clinical practice.

## Figures and Tables

**Table 1:** Examples of deregulated levels of circulating miRNAs in various malignancies proposed to have either diagnostic and/or prognostic value. MDS: Myelodysplastic syndrome; HCC: Hepatocellular carcinoma; MM: Multiple myeloma; DLBCL: Diffuse large B-cell lymphoma; HL: Hodgkin lymphoma; CNS: Central nervous system; AML: Acute myeloid leukemia; CLL: Chronic lymphocytic leukemia; ATL: Adult T-cell leukemia; BAL: Bronchoalveolar lavage; D: Diagnostic; PG: Prognostic; and PD: Predictive of response.

Circulating miRNA	Cancer Type	Type of Biomarker	Body Fluid Type	Cohort Size		Reference
				Cases	Controls	
*let-7a*	Prostate	D	Blood	75	27	[52]
	Colorectal	D	Serum exosomes	88	11	[53]
		D	Plasma	51	26	[54]
	MDS	PG	Plasma	50	76	[55]
	Gastric	D	Plasma	69	30	[56]
*let-7a/b*	Lung	D, PG	Serum/plasma	220	220	[57]
*let-7b*	HCC	D, PG	Serum	120	30	[58]
	Ovarian	D	Serum	18	12	[59]
*let-7c*	Lung	D	Plasma	20	360	[60]
	Breast	D	Serum	90	64	[61]
*let-7c/i/f*	Gastric	D	Serum	214	424	[62]
*let-7d*	Prostate	PG	Plasma	50	10	[63]
*let-7e*	MM	PG	Serum	121	30	[64]
	Thyroid	D	Serum	95	44	[65]
*let-7f*	Ovarian	D, PG	Plasma	360	200	[66]
	Colorectal	D	Feces	51	26	[54]
	Lung	D, PG	Plasma vesicles	106	68	[67]
	HCC	D, PG	Serum	90	60	[68]
*let-7i*	Lung	PG	Serum	10	20	[69]
	Ovarian	D	Serum/plasma	25	25	[70]
*miR-10*	Breast	D, PG	Serum	113	-	[71]
		PG	Serum	89	29	[72]
		PG	Cerebrospinal	16	15	[73]
	Lung	D	Serum	42	28	[74]
		PG	Cerebrospinal	28	15	[73]
	Glioblastoma	D	Cerebrospinal	19	15	[73]
	Oesophageal	D	Serum	50	50	[75]
*miR-16*	Oral	D	Serum	30	26	[76]
	Prostate	D	Serum	21	15	[77]
		D	Serum	73	20	[78]
	Breast	D	Serum	76	76	[79]
	Osteosarcoma	D	Serum	20	20	[80]
	Gastric	D, PG	Plasma	30	18	[81]
		D, PG	Serum	50	47	[82]
	Liver	D	Serum	90	60	[68]
	Oesophageal	D, PG	Plasma	38	19	[83]
*miR-17*	Gastric	D, PG	Serum	50	47	[82]
	Liver	D	Serum	90	60	[68]
	Oesophageal	D, PG	Plasma	38	19	[83]
*miR-18a*	Oesophageal	D	Serum	106	54	[84]
	Gastric	D	Plasma	104	65	[85]
	Breast	D, PD	Serum	108	75	[86]
	Colorectal	D	Stool	198	198	[87]
*miR-17/19a*	Melanoma	PD	Plasma	13	13	[88]
*miR-19a*	Breast	PD	Serum	30	38	[89]
		D	Serum	63	21	[90]
		D, PG	Serum	113	-	[71]
	Bladder	D	Plasma	50	50	[91]
	Colorectal	D, PG	Serum	90	12	[92]
	MM	D,PG,PD	Serum	108	56	[93]
	Lung	D, PG	Serum	201	103	[94]
*miR-19b*	Gastric	D, PG	Plasma	30	18	[81]
	Lung	D, PG	Serum	94	94	[95]
*miR-20a*	Prostate	D	Plasma	82	-	[96]
	Lung	D	Plasma	126	60	[97]
	Osteosarcoma	D	Serum	20	20	[80]
	Colorectal	D	Feces	397	198	[98]
	Esophageal	D	Plasma	70	40	[99]
*miR-17/-92*	Colorectal	PD	Serum	37	7	[100]
	Colorectal	D	Plasma	90	90	[101]
*miR-92*	Ovarian	D	Serum	28	15	[102]
*miR-92a*	Colorectal	D	Plasma	152	75	[103]
	Leukemia	D	Plasma	77	16	[104]
*miR-92a/b*	Prostate	D	Serum	21	15	[77]
*miR-106a*	Gastric	D, PG	Plasma	48	22	[105]
	Colorectal	D	Feces	117	107	[106]
		D	Plasma	100	79	[107]
		PG	Serum	175	130	[108]
*miR-17/-106a/b*	Gastric	D	Plasma	69	30	[56]
*miR-17/-106b*	Gastric	D	Serum	72	36	[109]
*miR-106b*	Liver	D	Plasma	47	61	[110]
		D	Serum exosomes	20	40	[111]
	Breast	D, PG	Plasma	173	50	[112]
	Gastric	D, PG	Plasma	20	20	[113]
	Bladder	D	Urine	112	78	[114]
	Ovarian	D	Serum	31	31	[115]
*miR-21*	DLBCL	D	Serum	60	43	[11]
		D,PD,PG	Serum	112	45	[116]
		D	Serum	60	43	[11]
		D,PD,PG	Serum	62	50	[117]
	HL	D	Plasma	42	20	[118]
	CNS lymphoma	D, PG	Serum	37	53	[119]
	Breast	D	Plasma	14	8	[120]
		D, PG	Serum	62	10	[121]
		D, PG	Serum	30	60	[122]
		D, PG	Serum	50	82	[123]
		PG	Serum	326	223	[124]
		D	Plasma	114	116	[125]
		D	Urine	24	24	[126]
		PG	Serum	113	-	[71]
	Gastric	D	Plasma	69	30	[56]
		D, PG	Plasma	42	-	[127]
		PG	Serum	103	-	[128]
		PG	Serum	79	-	[129]
		PG	Serum	64	64	[130]
		PG	Plasma	69	-	[131]
		D	Serum	50	50	[132]
	Glioblastoma	PG	Serum	30	30	[39]
		D	Plasma	10	10	[133]
	Ovarian	D	Serum	28	15	[102]
		D, PG	Serum	94	40	[134]
		D	Serum	60	10	[135]
	Pancreatic	D	Plasma	49	36	[136]
		D, PG	Plasma	32	30	[137]
		D	Plasma	24	24	[138]
		D	Stool	30	15	[139]
		D	Serum	22	14	[140]
		D	Plasma	30	26	[141]
		D	Saliva	7	4	[142]
	Prostate	PD	Plasma	82	-	[96]
		PD	Serum	56	-	[143]
	Colorectal	PD	Serum	37	7	[100]
		D, PG	Serum	40	40	[144]
		D	Serum	160	77	[144]
		D, PG	Serum	186	96	[145]
		D	Serum	200	130	[146]
		PG	Serum	102	-	[147]
		D	Serum	56	197	[148]
	Melanoma	PD	Plasma	13	13	[88]
	Lung	D, PG	Plasma	25	25	[97]
		D, PG	Serum	152	300	[149]
		D	BAL + sputum	21	10	[150]
		D, PG	Serum	80	60	[151]
	Neck	D	Plasma	50	36	[152]
	Oesophageal	D	Plasma	50	20	[153]
		D	Saliva	39	19	[154]
		D, PG	Plasma	38	19	[83]
	Liver	D	Serum	52	43	[155]
		PG	Serum	224	-	[156]
		D, PG	Serum	30	60	[157]
		D	Serum	23	17	[158]
	Biliary tract	D	Plasma	94	73	[159]
	Nasopharyngeal	D	Plasma	217	73	[160]
	Osteosarcoma	D, PG	Plasma	40	40	[161]
*miR-29a/b/c*	Osteosarcoma	D, PG	Serum	80	80	[162]
*miR-29a*	Colorectal	D	Plasma	152	75	[103]
		D	Serum	38	36	[163]
		D	Serum	30	26	[164]
	Breast	D	Serum	63	90	[165]
		D	Serum	20	20	[166]
	Oral	D	Serum	30	26	[76]
	Ovarian	D	Serum	28	15	[102]
*miR-29b*	Colorectal	D, PG	Serum	55	55	[167]
*miR-29c*	Colorectal	PG	Serum	103	37	[168]
	Lung	D	Serum	70	48	[169]
	Nasopharyngeal	D	Serum	160	143	[170]
*miR-30a*	Lung	D	Plasma	60	75	[171]
	Esophageal	D	Serum exosomes	18	29	[172]
*miR-30c*	Prostate	D	Plasma	105	115	[173]
		D	Plasma	59	27	[174]
	Lung	D	Serum	80	40	[175]
*miR-30d*	Lung	PG	Serum	82	50	[176]
		PG	Serum	303	-	[177]
*miR-30e*	Liver	D	Serum	39	31	[178]
*miR-34a*	Breast	D, PG	Serum	89	29	[74]
	Lung	D	Blood	22	27	[179]
*miR-34b*	Prostate	D	Serum	21	15	[77]
	Osteosarcoma	D	Plasma	133	133	[180]
*miR-34b/c*	Breast	D	Serum	15	15	[181]
*miR-34c*	Lung	D	Serum	17	19	[182]
*miR-125a*	Oral	D	Saliva	50	62	[183]
	Lung	D	Serum	70	70	[184]
	Breast	PG	Serum	300	-	[185]
	Liver	PG	Serum	120	255	[186]
*miR-125b*	Breast	PD	Serum	56	10	[187]
		D	Plasma	197	142	[125]
	Lung	D, PG	Serum	193	110	[188]
		PG, PD	Serum	260	260	[189]
	Oral	D	Plasma	85	46	[190]
	Colorectal	D	Serum	160	77	[191]
	Glioma	D	Serum	33	33	[192]
	Melanoma	D	Serum exosomes	21	35	[193]
*miR-155*	Breast	D	Serum	63	21	[90]
		D	Plasma/serum	184	75	[194]
		D	Serum	20	10	[195]
		D	Serum	103	55	[196]
		D, PG	Serum	89	29	[74]
		PG	Serum	32	120	[197]
	Colorectal	D, PG	Serum	146	60	[198]
	Lung	D	Serum	36	32	[199]
		D	Serum/plasma	220	220	[57]
	Esophageal	D, PG	Plasma	60	60	[200]
	AML	D	Serum	140	135	[201]
	DLBCL	D	Serum	75	77	[202]
		D	Serum	60	43	[11]
	CLL	PG, PD	Plasma	228	-	[203]
	ATL	PG	Plasma	35	-	[204]
*miR-200a/b/c/141*	Ovarian	D	Serum exosomes	50	20	[135]
*miR-200a/b/c*	Ovarian	D, PG	Serum	70	70	[205]
		D	Serum	28	28	[206]
*miR-200c/141*	Breast	D, PG	Blood	57	20	[207]
*miR-200c/141*	Ovarian	D, PG	Serum	93	50	[208]
*miR-200a*	Oral	D	Saliva	50	62	[183]
*miR-200b*	Prostate	PG, PD	Serum/plasma	97	-	[209]
*miR-200c*	Colorectal	D	Plasma	78	86	[210]
		PG	Serum	206	24	[211]
	Gastric	D, PG	Serum	98	100	[212]
		D, PG	Blood	52	15	[213]
	Lung	D, PG	Serum	70	44	[214]
	Esophageal	PG, PD	Serum	64	-	[215]
*miR-141*	Urinary tract	D	Serum	44	34	[216]
	Lung	D	Serum	42	28	[74]
	Prostate	D	Serum	25	25	[12]
		PG	Serum	113	-	[217]
		D	Serum	21	15	[77]
		D	Plasma vesicles	78	28	[218]
		D	Serum exosomes	71	80	[219]
		PG	Serum	30	26	[220]
	Colorectal	PG	Plasma	185	76	[221]
*miR-429*	Lung	D, PG	Serum	70	48	[169]
*miR-210*	DLBCL	D	Serum	60	43	[11]
	Pancreatic	D	Plasma	49	36	[136]
		D	Plasma	22	25	[222]
	Renal	D	Serum	78	42	[223]
		D	Serum	34	23	[224]
	Breast	PG, PD	Plasma	69	43	[225]
	Pancreatic	D	Saliva	7	4	[142]
		D, PG	Pancreatic juice	6	6	[226]
		D	Plasma	30	26	[141]
	Bladder	D, PG	Serum	168	104	[227]
		D	Urine	94	56	[228]
	Glioma	D, PG	Serum	136	50	[229]
	Liver	PD, PG	Serum	113	39	[230]
*miR-221*	Colorectal	D	Plasma	103	37	[231]
		D	Stool	198	198	[87]
	Prostate	PG	Plasma	82	-	[96]
	Leukemia	D	Plasma	79	37	[232]
	Liver	D	Serum	20	40	[111]
	Larynx	D	Plasma	30	30	[233]
	Glioma	D, PG	Plasma	50	51	[234]
	Melanoma	D, PG	Serum	72	54	[235]
	Renal	PG	Plasma	77	-	[236]
*miR-375*	Breast	PG	Serum	68	-	[237]
	Lung	D, PG	Plasma	217	217	[238]
		PG	Serum	113	-	[217]
	Prostate	PG	Serum	47	72	[218]
		PG	Serum	84	-	[239]
		PG	Plasma	100	-	[240]
		D	Plasma	78	28	[218]
	Oesophageal	D	Plasma	38	19	[83]
		PG	Serum	194	94	[241]
		D	Plasma	50	20	[153]
	Liver	D	Serum	78	156	[242]
	Colorectal	D	Plasma	88	40	[243]
